# One-Year Functional Outcomes of Medial Congruent, Manually Instrumented Total Knee Replacements

**DOI:** 10.7759/cureus.104403

**Published:** 2026-02-27

**Authors:** Jonathan Courtney

**Affiliations:** 1 Department of Surgery, Florida Atlantic University Charles E. Schmidt College of Medicine, Boca Raton, USA

**Keywords:** koos jr, koos outcomes, mechanical axis alignment, medial-congruent bearing, total knee replacement (tkr)

## Abstract

Aim: The primary objective of this study was to evaluate improvement in Knee Injury and Osteoarthritis Outcome Score for Joint Replacement (KOOS-JR) following mechanically aligned, manually instrumented total knee arthroplasty (TKA) using a medial congruent design. Secondary objectives included assessment of complication rates, reoperations, and revision-free survivorship at one year.

Methods: A retrospective chart review was conducted of 200 consecutive primary TKAs performed by a single surgeon. The cohort included 90 male and 110 female patients with a mean age of 73 years at the time of surgery. All procedures were performed using manual instrumentation with mechanical alignment targets and a medial congruent polyethylene design. Patient-reported outcomes were assessed using the KOOS-JR preoperatively, at six weeks postoperatively, and at approximately one year following surgery. Complications, reoperations, and revision procedures were recorded. Descriptive statistics were used to summarize outcomes and complication rates.

Results: The mean preoperative KOOS-JR score was 56.2. At six weeks postoperatively, the mean KOOS-JR score improved to 78.3, demonstrating substantial early functional improvement. At an average follow-up of one year, the mean KOOS-JR score further improved to 89.4, indicating sustained and clinically meaningful gains in pain relief and function. No revision surgeries were required during the study period, resulting in 100% implant survivorship at the latest follow-up. Six patients (3%) required manipulation under anesthesia for postoperative stiffness. No cases of early aseptic loosening, mechanical failure, or implant-related instability were identified.

Conclusions: In this retrospective series, mechanically aligned primary TKA performed with manual instrumentation and a medial congruent implant design resulted in significant improvements in patient-reported outcomes, low complication rates, and excellent early implant survivorship. The observed improvements in KOOS-JR scores exceeded established thresholds for clinical significance and were maintained at one year postoperatively. These findings support the effectiveness and reproducibility of this surgical approach for primary TKA and suggest that favorable outcomes can be achieved without the routine use of advanced alignment technologies. Longer-term follow-up and comparative studies are warranted to confirm durability and assess outcomes relative to alternative alignment strategies.

## Introduction

Total knee arthroplasty (TKA) is the definitive intervention for alleviating pain and restoring function in patients with end-stage knee osteoarthritis [1]. Conventional mechanically aligned TKA, performed with manual instrumentation, remains the predominant global surgical approach due to its established long-term outcomes and reproducibility [1]. More recently, technology-assisted procedures, such as robotic-assisted TKA (RA-TKA), have gained attention for potentially improving surgical accuracy. However, the clinical superiority of these advanced technologies in routine practice remains a subject of ongoing debate. Recent systematic reviews comparing RA-TKA to manual techniques consistently indicate that while robotic assistance may enhance alignment precision, this metric often does not translate into clear or consistent benefits in patient clinical and functional outcomes [2-4].

The primary objective of this single-surgeon, single-institution study was to evaluate improvement in patient-reported outcomes, specifically Knee Injury and Osteoarthritis Outcome Score for Joint Replacement (KOOS-JR) scores, following mechanically aligned, manually instrumented TKA. Secondary objectives included assessment of complication rates, reoperations, and revision-free survivorship at one year. We hypothesized that conventional mechanically aligned TKA would result in significant improvement in KOOS-JR scores and that a high percentage of patients would achieve the Patient Acceptable Symptom State (PASS), defined as a KOOS-JR score of 71 or above [5], with complication rates comparable to those reported in RA-TKA series.

## Materials and methods

Study design and setting

This retrospective chart review included 200 patients who underwent primary, mechanically aligned TKA between 2023 and 2024 by a single fellowship-trained arthroplasty surgeon.

Patient selection

This study included patients who underwent TKA in 2023 and 2024. Inclusion criteria were patients undergoing primary unilateral TKA for osteoarthritis with available preoperative and postoperative KOOS-JR scores obtained up to one year following surgery, as well as the use of a medial pivot-type implant. Exclusion criteria included revision TKA, inflammatory arthritis, bilateral TKA, incomplete preoperative or postoperative KOOS-JR data, or the use of a posterior-stabilized implant.

Surgical technique

All procedures were performed using conventional mechanical alignment instrumentation with the Zimmer Persona Total Knee System (Zimmer Biomet, Warsaw, IN, USA). The alignment target for the tibial component was 90° to the mechanical axis. The alignment target for the femur was 5° for varus knees and 4° for valgus knees. All femoral components were cruciate-retaining, and medial congruent polyethylene inserts were utilized in all cases. The patella was not resurfaced in any patient; however, a lateral facetectomy was routinely performed.

The fixation method (cemented versus cementless) was determined intraoperatively based on bone quality and surgeon assessment.

All patients underwent general anesthesia with an adductor canal block administered by the anesthesia team. Postoperative analgesia followed a standardized multimodal pain management protocol.

Postoperative rehabilitation was standardized for all patients. Patients were mobilized on the day of surgery or postoperative day one and permitted weight-bearing as tolerated. All patients received structured home health physical therapy focusing on early range of motion, quadriceps activation, and gait training. A formal outpatient physical therapy referral was not routinely utilized. Manipulation under anesthesia was considered for patients who failed to achieve functional range of motion despite adherence to the prescribed rehabilitation program.

Data collection

Institutional Review Board (IRB) approval for exemption was obtained prior to data collection (IRB Solutions #0974). Patient demographics (age, sex, body mass index) and clinical data (complications, re-operations, and revisions) were extracted from electronic medical records. The KOOS-JR was collected preoperatively, at six weeks postoperatively, and at one year postoperatively. The KOOS-JR is a validated seven-item questionnaire focusing on morning stiffness, pain with various activities, and function within the past week. It uses a four-point scale from “none” to “extreme” to determine a general picture of knee health and disability [6]. The KOOS-JR is the preferred patient-reported outcome measure (PROM) for our practice because it is a valid and reliable tool for assessing knee physical capabilities while minimizing the burden on respondents [7]. The KOOS-JR, developed at Hospital for Special Surgery, is nonproprietary and free to use [8].

Statistical analysis

Descriptive statistics of frequencies, percentages, means, and standard deviations (SDs) were reported for all patient and surgical characteristics when appropriate. The KOOS-JR scores were reported as means and SDs at preoperative, six-week, and one-year post-op. Differences in outcomes across the follow-up timeframe were compared using repeated measures analysis of variance (ANOVA). The level of significance was set at a p-value of < 0.05. Statistical analyses were performed using Microsoft Excel (Microsoft, Redmond, WA, USA).

## Results

Patient demographics

Of the 353 total knee replacements that were performed during this time period, 200 patients met the inclusion criteria. All included patients had complete preoperative, six-week, and one-year KOOS-JR data. The remaining patients were excluded due to bilateral surgery (51 patients), the use of posterior-stabilized implants (37 patients), or a lack of one-year KOOS-JR scores (65 patients). Patient characteristics are summarized in Table 1. There were 90 males and 110 females with a mean age of 73 (range 45-90). The mean body mass index (BMI) was 28.8 (range 19-44). There were 169 cemented knees and 31 cementless knees.

**Table 1:** Patient Demographics SD, Standard Deviation; BMI, Body Mass Index

Variable	Value
Total, n	200
Mean age, yrs (range; SD)	73.0 (45 to 90; 9.1)
Sex, n (%)	
Female	110 (55)
Male	90 (45)
Mean BMI, kg/m² (range; SD)	28.8 (19.4 to 43.9; 5.3)
Laterality, n (%)	
Right	109 (55)
Left	91 (45)
Cement, n (%)	
Cemented	169 (85)
Cementless	31 (15)

Patient-reported outcomes

**Figure 1:**
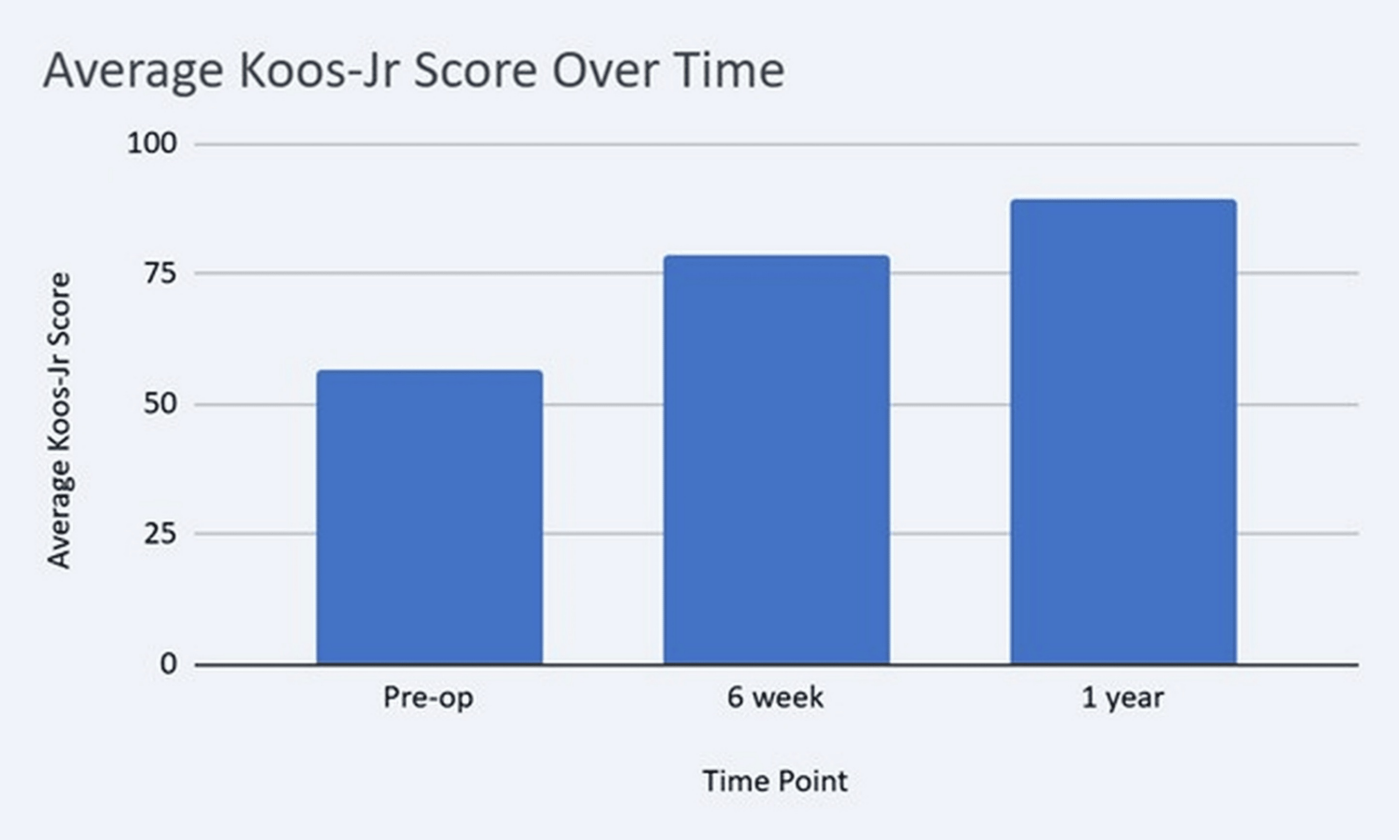
The KOOS-JR score improved significantly from 56.17 preoperatively, to 78.32 at six-week post-op, and 89.4 at one-year post-op. KOOS-JR: Knee Injury and Osteoarthritis Outcome Score for Joint Replacement [6]

**Figure 2:**
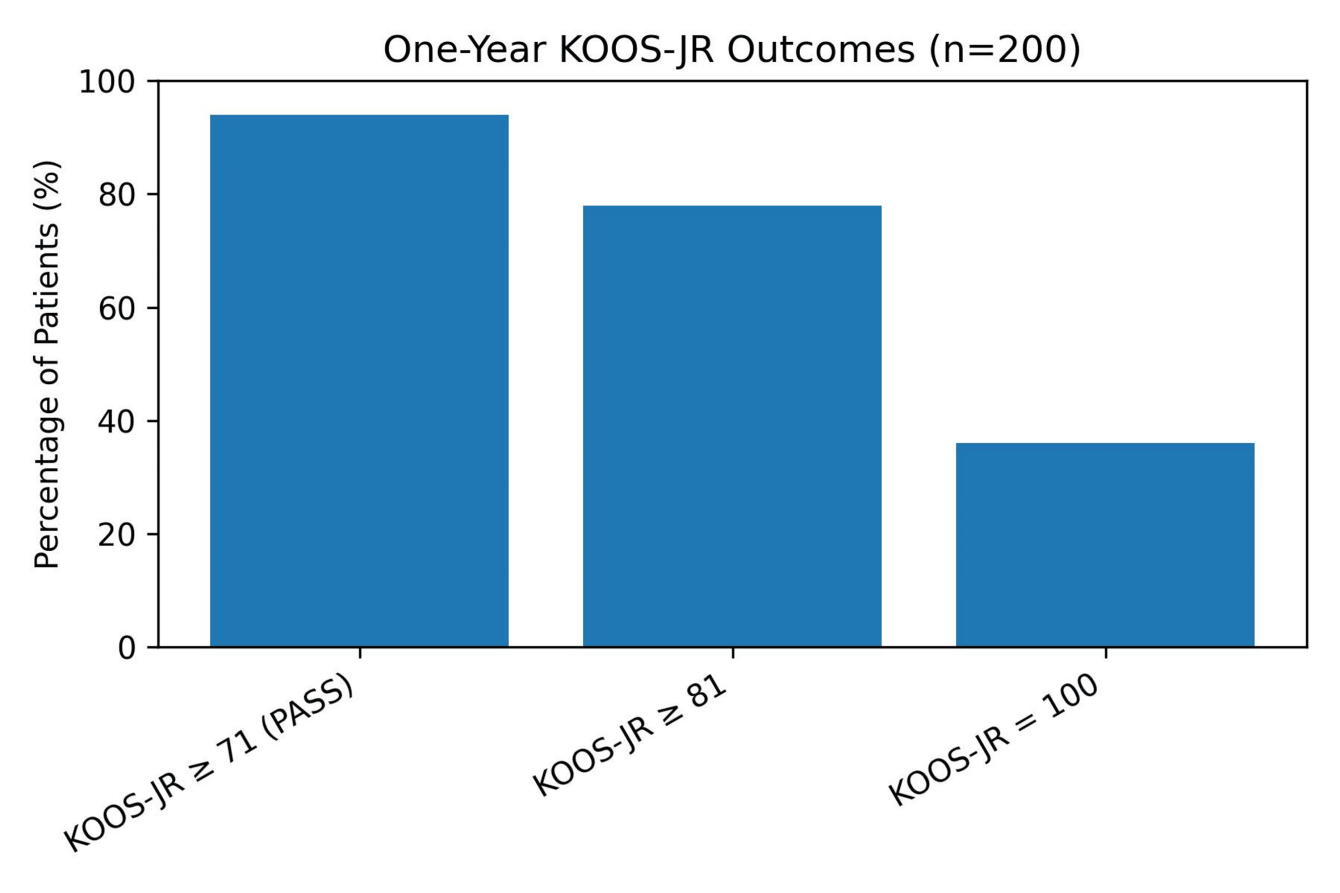
Ninety-four percent of patients achieved a KOOS-JR score of 71 or above (PASS), 78% of patients achieved a score of 81 or better, and 36% of patients achieved a perfect KOOS-JR score of 100 at one year. PASS: Patient Acceptable Symptom State [5], KOOS-JR: Knee Injury and Osteoarthritis Outcome Score for Joint Replacement [6]

KOOS-JR scores improved significantly from a mean of 56.17 (SD 10.66) preoperatively to 78.32 (SD 12.44) at six weeks and 89.4 (SD 10.38) at one year (Figure 1). One hundred eighty-eight out of 200 (94%) patients achieved a one-year KOOS-JR score of 71 or more. One hundred fifty-six out of 200 (78%) patients achieved a one-year KOOS-JR score of 81 or more. Seventy-two out of 200 (36%) achieved a one-year KOOS-JR score of 100 (Figure 2). Post hoc pairwise comparisons with Bonferroni correction confirmed significant improvement between all time points (p < 0.001).

Clinical outcomes

No revisions or infections were reported. Six patients (3%) underwent manipulation under anesthesia for postoperative stiffness. Two-patients had sustained patella fractures after falls, which were both treated non-surgically and healed without complication.

## Discussion

This study demonstrates that mechanically aligned, medial congruent design TKA provides substantial and clinically significant improvements in patient-reported outcomes, with a low incidence of adverse events. The improvements in KOOS-JR scores observed by six weeks and sustained at one year are meaningful to patients and consistent with high-quality outcomes reported following various TKA techniques [2-4].

For Medicare quality reporting under Merit-based Incentive Payment System (MIPS) following TKA, the PASS for KOOS-JR is defined as a score of ≥71 at one year postoperatively. This threshold represents a patient-reported functional outcome considered satisfactory by patients themselves [5].

In our cohort, 94% of patients (188 of 200) achieved or exceeded this PASS threshold at one year postoperatively. This rate compares favorably with published literature reporting PASS achievement rates generally ranging from approximately 80-90% following contemporary TKA, including studies evaluating robotic-assisted techniques [9]. These findings suggest that a consistent, mechanically aligned, manually instrumented approach can reliably produce excellent patient-reported functional outcomes without the added cost and complexity of advanced technologies.

Importantly, these results compare favorably with outcomes described in recent RA-TKA literature. Several systematic reviews and comparative studies show RA-TKA yields improved alignment metrics but mixed effects on PROMs, with many reporting similar functional scores at short to mid-term follow-up between robotic and conventional cohorts [2-4]. A recent systematic review published by Gonzalez et al. concluded that while RA-TKA is effective and safe, clinical outcomes are not universally superior to conventional methods [10]. Such findings suggest that the potential advantages of advanced technology, including perceived improvements in accuracy, do not consistently result in superior patient outcomes within the first year postoperatively. Samuel et al. performed a propensity-matched cohort study of 255 manual TKA and 85 RA-TKA patients at one year postoperatively, and found statistically equivalent PROMs and increased operating time in the RA-TKA cohort (113 minutes vs 105 minutes; p < 0.001) [11].

From a practical standpoint, manual mechanically aligned TKA offers advantages in terms of operative simplicity, lower direct costs, and broader accessibility. Robotic and computer-assisted platforms require significant capital investment, maintenance, and training, which may not be feasible in all practice environments and have not yet consistently demonstrated cost-effectiveness when considering similar PROMs outcomes in large cohorts. In a study comparing robotic versus manual TKA performed by high-volume surgeons, Tomkins et al. showed that total cost per case was nearly 40% higher and length of surgery was over 10% higher for robotic TKA than manual TKA without any differences in length of stay or complications [12].

The medial congruent (MC) bearing was designed to recreate more physiologic knee kinematics. In theory, having more normal knee kinematics would lead to better patient outcomes. In a prospective study comparing MC knees with posterior stabilized (PS) and cruciate retaining (CR) knees, Frye et al. found that an MC bearing provided similar or improved early pain, range of motion, KOOS scores, and patient satisfaction as compared with standard bearings [13].

This study also reinforces the notion that patella resurfacing during TKA is not necessary to achieve high function and good pain relief. A large-meta analysis of 7,075 TKAs has shown no significant difference in anterior knee pain in resurfaced and unresurfaced patella following TKA [14]. In a prospective, randomized, double blind study, Barrack et al. found no differences in pain or function in patella resurfaced versus unresurfaced TKA [15]. Kuljic et al. found a statistically significant increase in KOOS scores in patients whose patella was not resurfaced compared to patients whose patella was resurfaced during medial congruent TKA [16]. 

Limitations of this study include its retrospective design, which inherently limits the ability to establish causal relationships and introduces the potential for selection bias. Although all consecutive eligible patients were included and complete one-year follow-up was achieved for the analyzed cohort, patients without available one-year KOOS-JR data were excluded, which may introduce follow-up bias.

The absence of a contemporary comparison group, such as a robotic-assisted cohort performed by the same surgeon during the study period, limits the ability to directly compare techniques while controlling for surgeon-specific variables. Additionally, potential confounding factors, including variability in patient comorbidities, activity levels, socioeconomic factors, and adherence to rehabilitation, were not independently controlled or stratified in the analysis.

The single-surgeon, single-institution setting enhances internal consistency but may limit external validity and generalizability to broader practice environments with varying surgical techniques, implant choices, and rehabilitation protocols. Finally, follow-up was limited to one year, precluding conclusions regarding long-term survivorship or durability of outcomes. Nonetheless, this cohort adds important real-world data reinforcing that conventional mechanically aligned TKA remains a reliable, clinically effective option. 

## Conclusions

Mechanically aligned, manually instrumented TKA using a medial congruent design resulted in significant and clinically meaningful improvements in KOOS-JR scores at one year, with 94% of patients achieving the PASS and low complication rates. 

The outcomes observed in this study are consistent with those reported in contemporary robotic-assisted TKA literature. However, given the retrospective design and absence of a direct comparison cohort, definitive conclusions regarding equivalence or superiority cannot be established. Rather, these findings demonstrate that mechanically aligned, manually instrumented TKA can reliably achieve excellent short-term patient-reported outcomes when performed using a standardized technique. In the context of value-based care, these data support the continued use of mechanically aligned TKA as a reproducible and effective surgical approach capable of delivering high-quality functional outcomes.
